# Dysfunction of Pre-Attentive Visual Information Processing in Drug-Naïve Women, But Not Men, During the Initial Episode of Major Depressive Disorder

**DOI:** 10.3389/fpsyt.2019.00899

**Published:** 2020-01-08

**Authors:** Xiuxian Yang, Qihe Wang, Zhengxue Qiao, Xiaohui Qiu, Dong Han, Xiongzhao Zhu, Congpei Zhang, Yanjie Yang

**Affiliations:** ^1^ Department of Medical Psychology, Public Health Institute of Harbin Medical University, Harbin, China; ^2^ Medical Psychological Institute, Second Xiangya Hospital, Central South University, Changsha, China; ^3^ The First Special Hospital of Harbin, Harbin, China

**Keywords:** pre-attentive processing, gender, major depressive disorder, visual mismatch negativity, cognitive function

## Abstract

Women are twice as likely as men to develop depression. Few studies have explored gender difference in cognitive function of patients with MDD. The gender difference in the pre-attentive information processing of MDD patients is still poorly understood. To examine the gender differences in change detection, 30 medication-free MDD patients (15 women) and 30 age and education matched controls (15 women) were recruited. The deviant-standard reverse oddball paradigm (50 ms/150 ms) was used to obtain the visual mismatch negativity (vMMN) in first episode MDD patients. Compared to men with MDD, women with MDD showed a significantly decreased increment vMMN, while no gender difference in decrement vMMN was found. The increment vMMN amplitude in MDD women was smaller than in healthy women, whereas no difference was found in decrement vMMN. Neither increment nor decrement vMMN differed between MDD men and healthy men. The mean amplitude of increment vMMN was not correlated with symptoms of MDD in MDD patients and MDD women. To conclude, the dysfunction of visual information processing existed at pre-attentive stage in MDD women.

## Introduction

Major depressive disorder (MDD) is one of the most common mood disorders and is a leading cause of disability worldwide (1). Women are twice as likely as men to suffer from MDD at some point in their life (2, 3). Moreover, depressed women experience more symptoms associated with MDD, and with greater severity, including sadness and somatic pain, compared to men (4, 5).

Although numerous studies have been conducted to explore the gender differences in the presentation and features of MDD, gender differences in cognitive function have been paid less attention. Several studies reported that cognitive functions were affected differently in depressed women compared to their male counterparts. The event-related potentials (ERPs) were widely used to measure the cognitive functions of some physical or mental stimuli. These potentials can be extracted from the ongoing electroencephalogram by filtering and signal averaging. P300, N170, MMN are all the ERP components which have been explored in cognitive function studies. Studies reported higher posterior P300 amplitude (reflect orientation of attention) in depressed patients, especially in women, indicating that the cognitive functions (e.g. automatic orientation of attention and controlled orientation of attention, response resolution, and working memory) of depressed women are more serious than those of depressed men (6–8). In addition, depressed women scored significantly lower in tests involving cognitive interference threshold (Stroop III) and visual recall (Rey-Osterreith Complex Figure Test) compared to depressed men (9), thus implying a more severe cognitive dysfunction in depressed women.

Pre-attentive information processing is the early stage of cognition which is essential for perception and cognition in humans. It is necessary for either survival or social skills development to filter information from pre-attentive processing to attentive processing (10). In addition, pre-attentive information processing provides effective basements for successful processing of advanced cognitive function such as task-relevant information (11). Moreover, the impairment of pre-attentive information processing may underline the clinical symptoms of some mental diseases (12, 13) and may regulate deficiencies in more complex cognitive processing (14). Hence, pre-attentive information processing has been widely used to study cognitive function in psychiatric patients (15).

Pre-attentive automatic detection can be indexed using visual mismatch negativity (vMMN), which reflects the difference waves between the ERPs elicited by deviant (presented infrequently) and standard (presented frequently) stimuli (16). The memory template was formed by the standard stimulus input repeatedly, when the deviant stimuli input against the template, the vMMN was generated. VMMN is a negativity deflection in the temporal-occipital electrodes during 150 ms and 350 ms after the deviant stimulus input. It can be produced by many different types of visual stimuli such as duration (17), color (18), motion direction (19), and facial expression (20). Importantly, vMMN has been explored in psychiatric and neural diseases, including Alzheimer’s disease (21), schizophrenia (22), and MDD (17, 20), indicating that vMMN can reflect the dysfunction of detecting the automatic changes of visual stimuli in patients with these diseases. Interestingly, reports on vMMN in patients with MDD have only been found in recent years. For example, Chang et al. found the amplitudes of vMMN decreased in MDD patients, reflecting a deficit in pre-attentive expression processing (20). In their study, the facial stimuli lasted for a period of time in the center of the screen, so it was difficult for participants to ignore facial expressions, although they were task-independent. Therefore, the vMMN in this study may not be a memory-comparison-base vMMN, which reflects automatic memory-based change. In support of the latter hypothesis, using a deviant-standard reverse paradigm, Qiu et al. reported that vMMNs which elicited by deviant duration were decreased in MDD patients. Obviously, the cognitive function of change detection in visual modality impaired in patients with MDD, however, the aforementioned studies matched patients and controls with gender, a direct interaction between depression and gender remains unknown.

Gender differences in automatic change detection in healthy participants have been explored and obtained inconclusive results so far. Using the traditional oddball paradigm, Langrova et al. found no gender differences in direction vMMN amplitudes (23), whereas our previous study reported that the amplitude of duration vMMN increased in males compared to females (24). The different findings may attribute to changes in previous research methods. The vMMN in Langrova et al.’s study was observed by traditional oddball paragram in which the vMMN is obtained by subtracting the ERP waveforms elicited by standard stimuli from that elicited by deviant stimuli (23), and hence the vMMN may have confounded low-level physical differences between deviants and standards. On the other hand, the standard stimuli are presented more frequently than the deviant stimuli in these studies, that is to say neuronal processing of the standard stimuli would likely have more refractory effects than those processing the deviant stimuli. Thus, the vMMN may not properly reflect the pre-attentive memory-based change detection because of the compound of refractory effect between the standard and deviant stimuli. Furthermore, our brain may process information differently for different physical properties of the stimuli (direction in Langrova vs. duration in Yang) (23).

In order to clarify gender differences in pre-attentive information processing in MDD patients, it is essential to obtain vMMN based on memory comparison. Schroger and Wolff designed a paradigm in which an equal-probability sequence protocol as a control condition to obtain a memory-comparison-base MMN (25). In particular, a deviant-standard-reverse oddball paradigm was introduced by Jacobsen and Schroger in which two stimuli were counterbalanced as standard or deviant in two separate blocks. And they found that the MMNs obtained under the deviant-standard inversion condition was similar to that observed under the control condition from the equal probability sequence, so they proposed to use the deviant-standard-reverse paradigm to obtain the memory-comparison-base MMN (26). In addition, it has reported that deviant stimuli could elicit memory-comparison-base MMN using the deviant-standard-reverse method with a “safe” presentation probability of 15% (27). Moreover, our previous studies have found that the visual and auditory pre-attentive information processing impaired in MDD patients by the reverse oddball paradigm with a presentation probability of 20% (17, 28). Therefore, we still employed the reverse oddball paradigm to acquire the memory-comparison-base vMMN in this study.

Based on the above studies, we hypothesized that the impairment of pre-attentive information processing was more severe in MDD women compared to MDD men. Therefore, the aim of the present study was to explore whether gender differences exist in the pre-attentive information processing among MDD patients, meanwhile to examine whether duration vMMN correlated with depressive symptoms.

## Methods

### Participants

Participants included 30 patients with MDD and 30 healthy controls. All participants had normal hearing and normal or corrected to normal vision. They were given a detailed procedure of the study, and written consent was approved and obtained by the Ethics Committee of the Harbin Medical University. The participants completing the experiment were paid seven dollars.

The MDD group consisted of 15 men and 15 women, which were recruited from the First Affiliated Hospital of Harbin Medical University. They all received structured interviews with the Diagnostic and Statistical Manual for Mental Illness (fourth edition, DSM-IV) to identify MDD. Before the ERP recording, all patients were interviewed by two psychiatrists to confirm the first episode and with no treatment. The 17-item Hamilton Rating Scale of Depression (HRSD-17) and the 14-item Hamilton Anxiety Rating scale (HAMA) were used to evaluate the severity of depression and anxiety respectively. Twenty out of the 30 MDD patients appeared anxiety symptoms. Patients were excluded if they had any other axis-I mental disorders.

The healthy controls (15 males and 15 females) were recruited from the Physical Examination Center in the First Affiliated Hospital of Harbin Medical University. The Structured Clinical Interview for DSM-IV (SCID) was also used to exclude any psychiatric disease, neurological illness, alcohol or drug abuse, and medications known to affect the test. HRSD-17 and HAMA were measured in control group as well. Education level and age matched between controls and patients. And the participants with traumatic brain injury and intellectual impairment were excluded in both groups.

An overall ANOVA with group and gender as between-subject factor was conducted to analyze age, education, depression score (HRSD-17), and anxiety score (HAMA) in MDD group and healthy control group. The main effect of group was found in depression score and anxiety score. No gender effect was found in age, education, depression score, and anxiety score. The interactions of Group × Gender on age, education, depression, and anxiety severity were not significant (**Table 1**).

**Table 1:** An overall ANOVA of demographic characteristic in MDD group and healthy control group (M, SD).

Group	Age (year)	Education (year)	Anxiety	Depression
MDD patients				
MDD men	40.95, 10.38	8.47, 5.08	14.69, 5.60	24.93, 4.65
MDD women	41.96, 9.91	8.53, 4.97	15.37, 6.56	25.67, 4.19
Healthy controls				
Healthy men	41.19, 10.50	8.47,4.24	2.08, 0.96	1.95, 0.72
Healthy women	41.27, 9.76	9.13,4.63	2.58, 1.12	2.16, 0.85
Group effect	F = 0.009, P = 0.925	F = 0.060, P = 0.807	F = 547.971, P = 0.000	F = 1.732.082, P = 0000
Gender effect	F = 0.269, P = 0.606	F = 0.090, P = 0.766	F = 0.003, P = 0.957	F = 1.511, P = 0.224
Group × Gender	F = 1.297, P = 0.263	F = 0.060, P = 0.807	F = 0.146, P = 0.704	F = 0.771, P = 0.384

### Stimuli and Procedure

**Figure 1:**
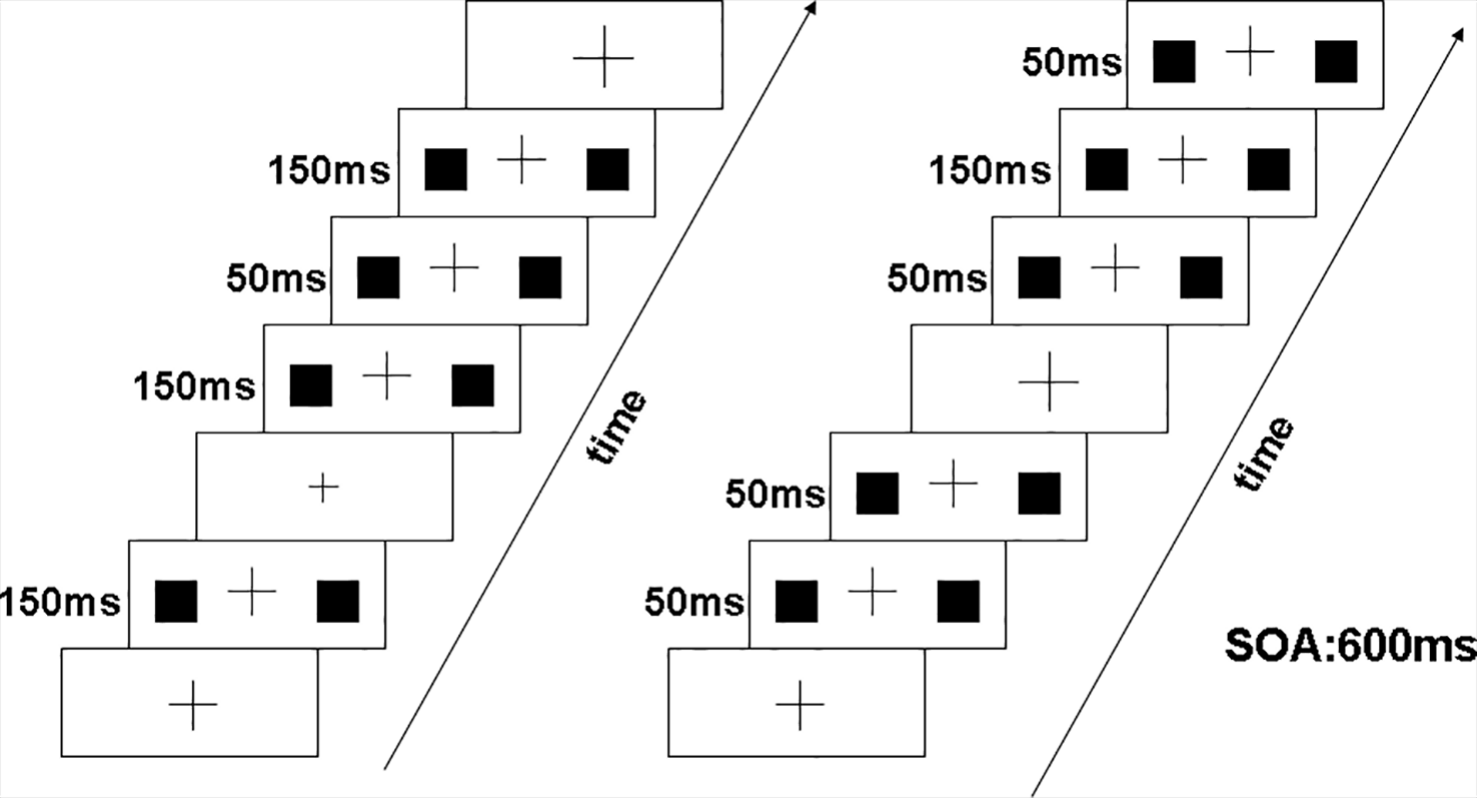
Visual stimuli consisted of two solid black squares (1 cm × 1 cm) presented simultaneously for 50 ms or 150 ms in the periphery. The left panel illustrates deviant stimuli at 50 ms and standard stimuli at 150 ms, and right panel illustrates deviant stimuli at 150 ms and standard stimuli at 50 ms.

Participants sat in a sound attenuated, electrically shielded dark room and were focused on a black cross presented in the center of a white screen with luminance of 56 cd/m^2^. The black cross was displayed continuously throughout the stimulus blocks. Response hands were counterbalanced across participants. As shown in **Figure 1**, at the beginning of the experiment, the cross was presented 1,000 ms. Occasionally, the cross become bigger or smaller randomly (mean frequency: 12/min). Two solid black squares (1/1 cm) were presented 50 or 150 ms at the same time in the periphery with a visual angle of 3.8° × 4.0°. The stimulus onset asynchrony (SOA) was fixed at 600 ms. When the peripheral squares were appeared, the cross was simultaneously presented with unchanged size. The duration of the bigger or smaller cross was 150 ms. Upon resizing of the cross, participants were requested to ignore the peripheral stimuli. And they pressed the left or the right button accordingly within 800 ms when the cross became “big” or “small.” Participants were inquired whether they noticed changes of the peripheral squares after finishing the experiment. No participant reported awareness of these changes.

Our experiment included two conditions: decrement deviant (50 ms duration) with a 150 ms standard (A) and increment deviant (150 ms duration) with a 50 ms standard (B). The decrement MMN was obtained by subtracting the ERP of decrement deviant in (A) from the ERP of the 50 ms presented as a standard in (B). Similarly, the increment MMN was calculated by subtracting the ERP of increment deviant in (B) from the ERP of 150 ms presented as a standard in (A). Under the two blocking conditions, the exposure probability of deviation (20%) and standard stimulus (80%) was equal, and the blocking order between subjects was balanced. Each block included six experimental sequences of 150 trials. In each condition, the stimuli were presented in pseudorandom manner with two or more standard stimuli before each deviant stimulus.

### EEG Recording

EEG readings were recorded continuously with a Neuroscan 40-electrode cap (NuAmps amplifier) including channels based on the International 10-20 system. The horizontal EOG was recorded by two electrodes placed on the outer canthi of both eyes and vertical EOG was recorded by another two electrodes placed above and below the right eye. vMMNs were recorded by the electrode sites O1, O2, and Oz placed on the occipital. The reference electrode was put on the tip of the nose. The impedances of all electrodes were less than 5 kΩ throughout the experiment and the sampling rate was 500 Hz/channel.

After correcting EOG artifact with the method of Gratton (29), the EEG was segmented to 500 ms including 100 ms pre-stimulus and 400 ms time locked to stimuli onset. We rejected the trials with artifacts greater than ±100 μV and the trails in which the participant responded. The EEG segments were averaged respectively for the deviant and standard stimuli and the averaged ERP data were digitally filtered with a band-pass filter at 1–30 Hz, 24 dB/octave. In MDD group, the numbers of accepted/rejected epochs for each stimulus were 83/4 and 332/13 (deviant, standard in 50 ms deviant condition), and 84/3 and 324/12 (deviant, standard in 150 ms deviant condition), respectively. In control group, the numbers of accepted/rejected epoch for each stimulus were 87/5 and 333/15 (deviant, standard in 50 ms deviant condition), and 86/4 and 329/11 (deviant, standard in 150 ms deviant condition), respectively.

### Data Analysis

The software Statistical Package for the Social Sciences (version 18.0 for Windows) was used to conduct statistical analyses. Repeated measures ANOVA with group and gender as between-subject factors were used to analyze the performance data (reaction times, correct rate). The mean amplitudes of the vMMN were measured during the time window of 150–300 ms. All the analysis were conducted on the average amplitudes of O1, O2, and OZ. Group and gender were used as the factors between the subjects, hem or location (O1/OZ/O2) were used as the factors within the subjects for 2*2 ANOVA. The Greenhouse-Geisser procedure was used to correct the statistical probability. Tukey’s honestly significantly different (HSD) test was used as a *post-hoc* test. A *p*-value of < 0.05 was considered statistically significant.

## Results

### Behavioral Performance

For detecting cross changes in the center of the screen, responses were scored as hit if the correct button was pressed within 150 to 800 ms after target onset. For accuracy rate, the main effect of group and gender on participants’ accuracy failed to reach significance (group: *F* = 0.848, df = 1, 56, *p* = 0.361, η^2^ = 0.015; gender: *F* = 0.053, df = 1, 56, *p* = 0.819, η^2^ = 0.001). And the group × gender interaction did not reach significance level (*F* = 0.205, df = 1, 56, *p* = 0.652, η^2^ = 0.004).

For reaction time (RT), the main effect of group (MDD group and control group) on reaction time reached significance (*F*_(1,__56)_ *=* 11.243, *p* = 0.001, η^2^ = 0.167), and the reaction time was longer in MDD group than control group (MDD group: 495.3 ± 9.12 ms; control group: 452 ± 10.0 ms). The main effect of gender failed to reach significance (*F*_(1,__56)_ *=* 0.827, *p* = 0.367, η^2^ = 0.015). The group × gender interaction did not reach significance level (*F*_(1,__56)_ *=* 0.306, *p* = 0.583, η^2^ = 0.005).

### ERP Analysis

**Figure 2:**
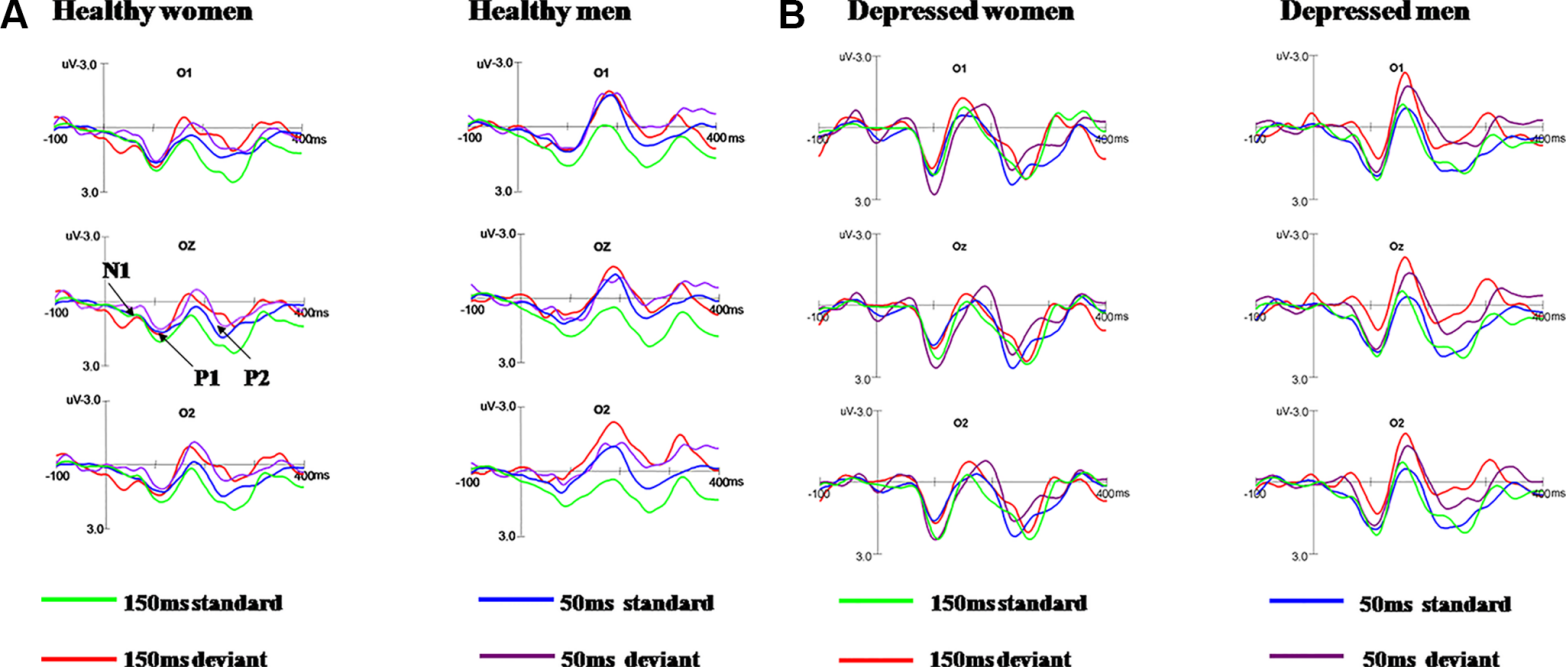
Grand average ERPs in response to 50 ms and 150 ms deviant/standard visual stimuli in healthy participants **(A)** and MDD patients **(B)**.

**Figure 3:**
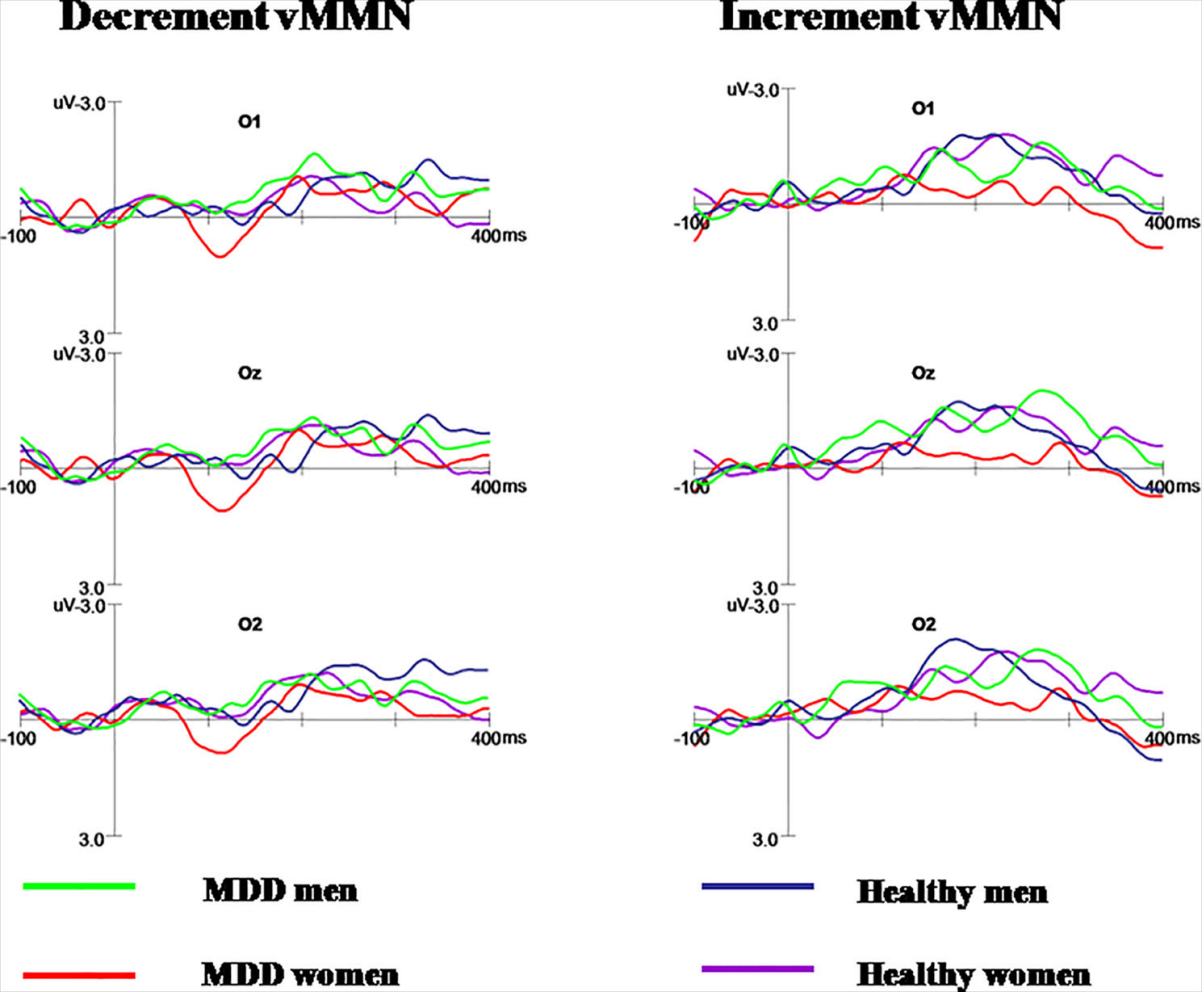
The decrement and increment vMMN in MDD men, MDD women, healthy men, and healthy women. MDD women showed significantly reduced increment vMMN compared to MDD men and healthy women. Increment vMMN was smaller in healthy women than in healthy men.

As shown in **Figures 2A**, **B**, N1 and P2 components elicited at the posterior scalp by the deviant and standard stimuli regardless of block conditions in both healthy and MDD participants. As illustrated in **Figure 3**, the vMMN was observed on occipital areas at the time range between 150 and 300 ms.

#### Increment vMMN

Repeated measures ANOVA showed the main effect of group on increment vMMN amplitudes in occipital areas was significant (F_(1,__56)_ = 6.130, p = 0.016, η^2^ = 0.099), indicating that the vMMN mean amplitude decreased in MDD group. The main effect of gender was marginal significant (F_(1,__56)_ = 4.980, p = 0.051, η^2^ = 0.034). Moreover, the interaction of group × gender reached significance (F_(1,__56)_ = 6.386, p = 0.014, η^2^ = 0.102).

**Figure 4:**
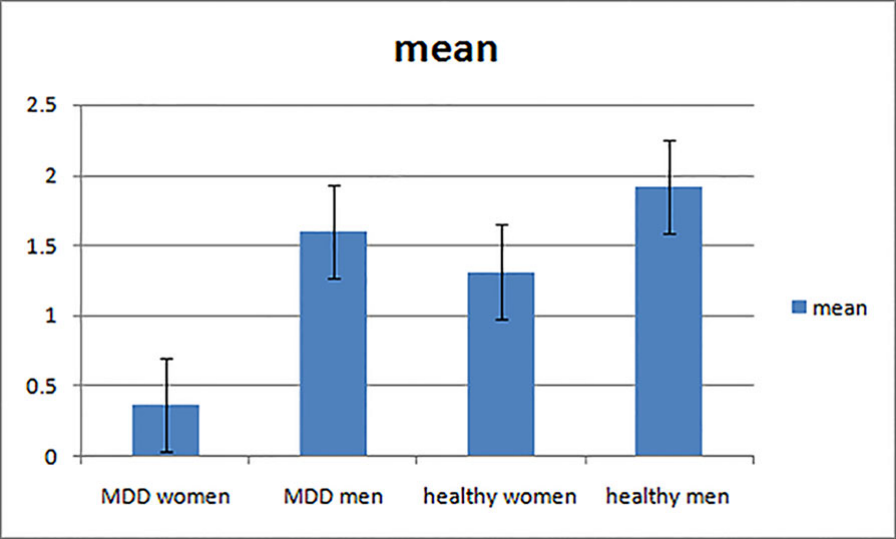
The mean amplitudes of increment vMMN in four groups.

Further post-hoc analysis revealed the amplitudes of increment duration vMMN in MDD women were significantly smaller compared to MDD men (−0.369 μV and −1.603 μV for MDD women and MDD men, respectively; *F*_(1,__56)_ = 6.696, *p* = 0.008). In addition, the increment vMMN in healthy men was higher than in healthy women (*F*_(1,__56)_ = 6.219, *p* = 0.016; healthy men: −1.916 μV, healthy women: −1.316 μV). The amplitude of increment vMMN was smaller in MDD women than in healthy women (−0.369 μV and −1.316 μV for MDD women and healthy women, respectively; *F*_(1,__56)_ *=* 4.603, *p* = 0.036) (**Figure 4**). No difference was found for increment vMMN between MDD men and healthy men (*F*_(1,__56)_ = 2.074, *p* = 0.155).

The main effect of hem and the interaction of hem × group × gender did not reach significance (hem: *F*_(2,__112)_ = 0.868, *p* = 0.387, η^2^ = 0.015; hem × group × gender: *F*_(2,__112)_ = 0.977, *p* = 0.353, η^2^ = 0.017).

#### Decrement vMMN

Repeated measures ANOVA showed no significant effect of group on decrement vMMN (F_(1,__56)_ = 0.240, p = 0.626, η^2^ = 0.004). The main effect of gender was not significant (F_(1,__56)_ = 0.557, p = 0.459, η^2^ = 0.010). Furthermore, there was no significant interaction for group × gender, indicating that the decrement vMMN amplitude was similar between different group for men and women. The main effect of hem was not significant (F_(2,__112)_ = 0.707, p = 0.495, η^2^ = 0.012). And the interaction of hem × group × gender did not reach significance (*F*_(2,__112)_ = 2.012, *p* = 0.153, η^2^ = 0.035).

### Relationship Between the Amplitudes of Increment vMMN and Depressive Symptom (Scores of HRSD) in MDD Patients

Pearson correlation analysis was used to examine the relationship between the amplitudes of increment vMMN and the severity of depression (scores of HRSD). At the occipital electrode sites, neither the correlation between vMMN amplitude and depression symptoms in the MDD patients (e.g., O1: r = 0.109, *p* = 0.678, Oz: r = 0.167, *p* = 0.302, and O2: r = 0.186, *p* = 0.396) nor the correlation in all participants was significant (e.g., O1: r = 0.126, *p* = 0.679, Oz: r = 0.149, *p* = 0.463, and O2: r = 0.201, *p* = 0.383). In addition, the amplitudes of the increment vMMN did not relate to the symptom level in MDD women (e.g., Oz, r = 0.185, *p* = 0.391, O1, r = 0.112, *p* = 0.634, and O2, r = 0.198, *p* = 0.367).

## Discussion

In this study, we examined gender differences in pre-attentive information processing using vMMN in MDD patients. Consistent with our hypothesis, we found that detection of change in vMMN was impaired in MDD women compared to MDD men. The results can be summarized as following: 1) In healthy controls, increment vMMN was enhanced in men relative to women. 2) In depressed patients, the amplitude of the increment vMMN was smaller in MDD women than healthy women. In contrast, MDD men showed no differences in increment vMMN amplitudes as compared to healthy men. 3) The amplitude of the decrement vMMN in MDD women was equivalent to healthy women, as was the case for MDD men and healthy men. 4) MDD women showed smaller amplitude of the increment vMMN compared to MDD men, indicating a greater severity of impairment in pre-attentive information processing in MDD women. In contrast, MDD women showed no differences in decrement vMMN amplitudes compared to MDD men. 5) The amplitude of increment vMMN did not associate with depressive symptoms in women with MDD, indicating the impairment of pre-attentive change detection in patients is independent of depressive symptoms.

In line with our previous study, the amplitude of vMMN in healthy males is higher than that in healthy females, which indicates that there are inherent gender differences in visual information processing (24).

To explain the gender difference in the increment vMMN, we must consider the neurogenerator of vMMN: the right occipital visual extrastriate area (30). It is reported that the visual region was sexually dimorphic by quantitative analysis of the cellular structure of human primary visual cortex. The primary visual cortex (V 1/Brodmann region 17) and the volume of the motion-sensitive region (hOc5) in men were larger than that of women (31), indicating that men have greater ability to process visual information. The enhanced VMMN observed in men in this study can indirectly demonstrate the gender differences in visual extrastriate areas. However, fMRI studies are needed to determine the gender differences in occipital visual extrastriate areas due to ERPs cannot directly reflect brain tissue structure.

Consistent with previous studies, MDD patients showed reduced vMMN compared to healthy controls, suggesting patients with MDD show impairment of pre-attentive processing. Our further analysis indicated that only MDD women exhibit smaller increment vMMN. The decreased vMMN indicates that impaired change detection function processing was evident in MDD women but not in MDD men. Although the neurobiological differences which may explain the gender-specific effect have not been elucidated, our results showed that male MDD patients differ from female patients in pre-attention information processing, which must be considered when comparing cognitive processing between MDD patients and healthy controls.

Although the amplitude of increment vMMN decreased in healthy women compared to healthy men and the same result in MDD women and MDD man, the amplitude decreased in MDD women compared to healthy women, while the amplitude in healthy man was similar to MDD men. Then we conclude that the pre-attentive change detection was impaired only in MDD women. And the results were mostly concordant with our hypothesis that there would be smaller vMMN amplitudes in MDD women than MDD men. Extensive studies have proved the role of personality factors in gender differences in depression and proposed the development of depression has been attributed to high levels of neuroticism (32, 33). Moreover, previous studies have found that levels of neuroticism were higher in MDD women compared with MDD men (34). In particular, MMN amplitude was positively correlated with neuroticism (35), and therefore it has been speculated that higher neuroticism may contribute to the difference of MMN between MDD women and MDD male.

It has been shown that MDD women make more errors during emotional processing tasks than healthy women and also MDD men (36). The differences in emotional processing between women and men during depressed states may result from different cognitive strategies regarding attention processing. In addition, studies have found that impairment of pre-attentive information processing may lead to more complex cognitive operations in patients with schizophrenia (14). Our findings that vMMN amplitude was smaller in MDD women compared to MDD men, as well as healthy women, suggests that gender differences in cognitive function during depressed states are related to the pre-attentive stage of information processing. Therefore, our results may partly explain disparities in the prevalence of MDD between women and men as well as the more severe impairments in cognitive function found in female patients. It is worth noting that given the link between depression and a narrow focus of attention, MDD women may have been impaired in detecting changes because their attention was more narrowly focused to the central task.

No significant effect for decrement vMMN was found between the MDD and healthy group, as well as between healthy men and women, while a significant effect was found for increment vMMN, which could be explained by different processing of increment and decrement vMMN. According to previous studies, stimulus parameters exert a differential influence on the vMMN for a duration decrement and increment of an equal magnitude. The increase in stimulus is detected by both a transient and a memory-comparison-based change detector system, while the decrease in stimulus is only activated by the change detector system (37, 38). The results of this study suggest that the impacts of stimulus parameters should be taken into account when comparing different studies in clinical settings.

Our previous studies indicated no association between the amplitudes of duration vMMN (17) or aMMN (28) and the severity of depressive symptoms in MDD patients. Furthermore, a few studies have reported that cognitive impairment remained evident regardless of the remission of depressive symptoms or the reduction of the HDRS score (39, 40). In the present study, the correlation between the severity of depressive symptoms and increment vMMN was not significant in MDD patients and MDD women, indicating that the pre-attentive change detection may be associated with depressive symptoms in patients with MDD. Taken together, our findings indicate the changes of vMMN are likely to be trait-dependent, but not state-dependent in MDD patients.

However, it should be noted that the cross-section design constrains conclusive evidence as to whether MDD affects pre-attentive processing or whether the impairment in pre-attentive processing is a risk factor for subsequently developing MDD in women. In addition, about 67% depressed patients have anxiety symptoms; our results could not exclude the effect of anxiety on our results. Moreover, Withall et al. found that compared with patients without melancholic symptoms, cognitive function of depressed patients with melancholy was severely impaired (41). Whether the subtypes of MDD affect the gender effect in pre-attentive processing needs further investigation. The evidence that symptom severity is not associated with vMMN suggests that the dysfunction of pre-attentive processing may be a significant trait of MDD but unrelated to the symptoms, and longitudinal data is required to verify whether vMMN abnormalities still exist if the depressive symptoms disappear. Last but not the least, the sample of our study is relatively small.

In conclusion, we found that the increment duration vMMN was reduced in MDD women but not in MDD men, indicating that MDD women have visual information processing dysfunction at the pre-attentive stage. Our study supports previous findings that cognitive impairment in MDD women was more severe than in MDD men. Therefore, we should take gender differences into account when exploring pre-attentive information processing in MDD patients. Moreover, the association between symptom severity and duration vMMN was not significant, reflecting that the dysfunction of pre-attentive processing was unrelated to the symptoms of MDD, it could be a distinguishing trait of MDD.

## Ethics Statement

This study was carried out in accordance with the recommendations of the Ethics Committee of Harbin Medical University with written informed consent from all subjects. All subjects gave written informed consent in accordance with the Declaration of Helsinki. The protocol was approved by the Ethics Committee of Harbin Medical University.

## Author Contributions

YY and XY designed the experiment. XY and QW wrote the manuscript. ZQ, XQ, DH, CZ, and QW selected the participants and completed the experiment. XZ and ZQ analyzed the data.

## Funding

This research was supported by the National Natural Science Foundation of China (Grant Nos. 81773536, 31271093, and 81302484) and National Science Foundation of Heilongjiang Province (Grant No: LH2019H032).

## Conflict of Interest

The authors declare that the research was conducted in the absence of any commercial or financial relationships that could be construed as a potential conflict of interest.
